# Supplementary material for: Ovine prenatal growth-restriction and sex influence fetal adipose tissue phenotype and impact postnatal lipid metabolism and adiposity in vivo from birth until adulthood
Source: PLoS One. 2020 Feb 14;15(2):e0228732. doi: 10.1371/journal.pone.0228732 (PMC7021317; doi:10.1371/journal.pone.0228732)
Supplement: Attachment — Submitted filename: PONE-D-19-32025 Response to Reviewers.docx [file pone.0228732.s003.docx]

PONE-D-19-32025 Response to Reviewers

Journal Requirements:

When submitting your revision, we need you to address these additional requirements.

1. Please ensure that your manuscript meets PLOS ONE's style requirements, including those for file naming.

Authors Response: Figure captions have been added to the figure ‘legends’ to meet journal requirements. As far as we are aware everything else is as per journal style

2. Please note that according to POS ONE policy, if materials, methods, and protocols are well established, authors may cite articles where those protocols are described in detail, but the submission should include sufficient information to be understood independent of these references (<https://journals.plos.org/plosone/s/submission-guidelines#loc-materials-and-methods).Thus>, we kindly request that you provide more information on the methods used (especially for embryo transfer) and detail the composition of the diet administered in your study.

Authors Response: this request seems to conflict somewhat with the statement re the detailed methods made by referee 2. Nevertheless, we have expanded the text as requested and altered the references.

For embryo transfer the text now reads: ‘For recipients, oestrus synchronisation commenced on Day -15 relative to the onset of oestrus (Day 0) by insertion of vaginal sponges containing 60mg medroxyprogesterone acetate (Veramix®, Intervet UK Ltd, Welwyn Garden City, Herts, UK). Thirteen days later (Day -2) at 14:00h, sponges were removed, and ovulation stimulated by i.m. administration of 600 iu pregnant mare serum gonadotrophin (PMSG, Intervet UK Ltd). For donor ewes, synchronisation commenced on Day -14, when vaginal sponges containing 40mg fluorogestone acetate (Chronogest®, Intervet UK Ltd) were inserted. Seven days later (Day -7) these sponges were removed and immediately replaced with fresh ones of the same type. A superovulation regime was commenced on Day -4 at 08:00h with the i.m. administration of 1.125mg ovine follicle-stimulating hormone (oFSH, Ovagen®, Immuno-Chemical Products Ltd, Auckland, New Zealand) immediately followed by 125μg IM cloprostenol (Estrumate®, Schering-Plough Ltd, Welwyn Garden City, Herts, UK), a synthetic prostaglandin-F2α. At 18:00h a second dose of oFSH was given together with 400iu PMSG. Further twice daily doses of oFSH were administered at 08:00h and 18:00h on the following three days (Day -3 to -1) and sponges were removed on Day -2 at 18:00h. The onset of oestrus was assessed by presenting females to vasectomised rams three times daily to ensure optimum synchrony between potential donor and recipient animals. On Day 0 at 16:00h, donor ewes underwent insemination directly into the uterine cavity under direct laparoscopic visualisation, as previously described [31]. In both studies this involved fresh semen, collected by artificial vagina from the same sire of proven fertility, and diluted 1:3 with phosphate-buffered saline. Approximately 0.3ml of diluted semen was deposited in each horn. Four days following insemination, multiple embryos were recovered from donor ewes at laparotomy using a standard surgical technique of retrograde flushing of each oviduct. This was achieved by advancing a fine glass cannula into the fimbria, injecting ovum culture media (ICN Biomedicals, Ohio, USA) warmed to 37^o^C into the lumen of the ipsilateral uterine horn ~ 5cm from the utero-tubal junction, and milking the media out through the cannula into a sterile glass embryo dish. Developmental stage and quality were assessed using a stereomicroscope and the embryos held at 33^0^C in fresh ovum culture media until transferred to recipients within 4h of recovery. Embryos of optimum quality and stage were synchronously transferred in singleton into the uteri of adolescent recipients using a laparoscopically-assisted technique. Ovulation rate was determined, and the embryo transferred into the tip of the uterine horn ipsilateral to the ovary with the greater number of corpora lutea’.

We have detailed the composition of the diet as follows: ‘Diet composition was 30% coarsely milled hay, 41.5% barley 17.5% Hipro soya, 10% molasses, 0.35% salt, 0.25% limestone, 0.25% dicalcium phosphate and 0.15% of a vitamin-mineral mix’ and retained the original reference.

 3. According to our submissions guidelines, titles should be specific, descriptive, and concise; in this case, we suggest that your title is modified to clarify that all experiments were carried in vivo, and to specify the animal model used.

Authors Response: Ovine has been added as the first word of the title. We have also added ‘in vivo from birth’ to the title to reinforce that these are whole animal studies with final endpoints measured following necropsy.

Reviewers' comments:

Reviewer's Responses to Questions

**Comments to the Author**

1. Is the manuscript technically sound, and do the data support the conclusions?

The manuscript must describe a technically sound piece of scientific research with data that supports the conclusions. Experiments must have been conducted rigorously, with appropriate controls, replication, and sample sizes. The conclusions must be drawn appropriately based on the data presented.

Reviewer #1: Yes

Reviewer #2: Yes

2. Has the statistical analysis been performed appropriately and rigorously?

Reviewer #1: Yes

Reviewer #2: Yes

3. Have the authors made all data underlying the findings in their manuscript fully available?

The [PLOS Data policy](http://www.plosone.org/static/policies.action#sharing) requires authors to make all data underlying the findings described in their manuscript fully available without restriction, with rare exception (please refer to the Data Availability Statement in the manuscript PDF file). The data should be provided as part of the manuscript or its supporting information, or deposited to a public repository. For example, in addition to summary statistics, the data points behind means, medians and variance measures should be available. If there are restrictions on publicly sharing data—e.g. participant privacy or use of data from a third party—those must be specified.

Reviewer #1: No See response to reviewer’s comment below

Reviewer #2: Yes

4. Is the manuscript presented in an intelligible fashion and written in standard English?

PLOS ONE does not copyedit accepted manuscripts, so the language in submitted articles must be clear, correct, and unambiguous. Any typographical or grammatical errors should be corrected at revision, so please note any specific errors here.

Reviewer #1: Yes

Reviewer #2: Yes

5. Review Comments to the Author

Please use the space provided to explain your answers to the questions above. You may also include additional comments for the author, including concerns about dual publication, research ethics, or publication ethics. (Please upload your review as an attachment if it exceeds 20,000 characters)

Reviewer #1: This study provides a robust and highly controlled model of prenatal growth-restriction obtained by maternal overnutrition to decipher the effects on adipose tissue development and selected gene expression with consequences for lipid metabolism and body composition. Rather than reporting a snapshot at a single age, serial measures across the life-course are provided. Most factors are kept constant in the adolescent sheep model (one sire, transfer of single high-quality embryos harvested from donors with a known nutritional and reproductive background into young primiparous adolescent recipients of equivalent age, weight and adiposity at conception), allowing the effects of prenatal nutrient supply and also of sex to be demonstrated.
Minor comments:
- species should be indicated in the abstract and title (ewes)

Authors Response: Ovine has been added to the title and ovine model to the abstract. In addition, ‘dams’ has been replaced by sheep in the abstract.

- it’s a pity that fetus-derived DLK1 expression was not include in the list of genes studied by qPCR (e.g., Nat Genet. 2016 Dec;48(12):1473-1480. doi: 10.1038/ng.3699.)

Authors Response: We agree that based on the mouse knockouts and human cohort studied by these authors that this may have been an interesting avenue to pursue. However, it is perhaps pertinent to note that our animal model generates small but healthy offspring as a consequence of major placental growth restriction, and we have previously shown that these growth restricted fetuses show normal fetal weight-specific metabolic responses to insulin and glucose during fetal hyperinsulinemic-euglycemic and hyperglycemic-euinsulinemic clamps (https://www.physiology.org/doi/full/10.1152/ajpendo.00294.2007)

- the authors claimed for data availability. However, in the main text, means and standard errors are provided, but individual data are not included in supplementary files or repository.

Authors Response: We originally felt that presenting a large amount of data as means and standard errors, our extensive statistical approach and numerous figures showing individual data points (scattergrams) were adequate based on our own prior experience with the journal and having consulted recent papers published by PLOS ONE. Nevertheless, we have now uploaded the relevant individual data for each of the two studies in two separate supplementary information files, one for each study (namely, S1 Table and S2 Table) and indicated the availability of this data in the text, line 303-304.

Reviewer #2: This paper contributes new findings in a field that is well-known for authors. Excellent discussion and results of fetal gene expression are very interesting. Authors propose future lines of work, including more tissues and time points. I encourage them to follow them. However, some parts could be improved to increase its readability simplifying the manuscript and focusing on main ideas.

The abstract has too much information in some sentences. I think focusing on key ideas in the abstract would improve its readability. Adding ‘ovine model’ would also be better.

Authors Response: ‘Ovine model’ has been added for clarification as requested. The abstract was originally 255 words long and we believe that it provides an accurate account of the background, aims, design and main outcomes of two complementary studies. By including a late gestation and extensive postnatal study it is inevitable that the manuscript is data-rich, but such an approach is rare and allows one phase of life to be related to the other. The abstract text contains little hard data other than birthweight differences and is focused on the main differences between groups and sexes. We believe it is important for abstracting purposes across databases that the key elements of both studies are retained and presented in the abstract without truncation.

Good and informative introduction. However, its last parts about authors’ models and a very long and detailed second goal remember Materials & Methods.

Authors Response: I am afraid that we do not understand what point the referee is making here.

M&M is a detailed section, but weighty. Using supplementary documents could be useful to increase readability. I also think it would be better to have diets in supplementary data than in another paper.

Authors Response: The individual method description was originally brief in all cases and already referred the reader to the relevant individual original publications (i.e. normal practice). As requested by the editor more information on composition of the single complete diet used in the study has been added to the methods, together with detail of the embryo recovery and transfer procedures.

I think using bold type in tables is a great idea. In Results, there are averages from Experiment 2 without SEM values.

Authors Response: we assume that this comment refers to the text in original lines 411 to 414. The SEM values have now been added and the individual data points are available in the supplementary file.

Further details:
Some keywords are also in the title.

Authors Response: Is this not allowed? In any case the indication is that keywords are optional.

In table 2, the meaning of symbols (*,ɤ, ß) within individual groups is difficult to understand only reading the table.

Authors Response: the purpose of the accompanying footnote it to explain the use of symbols within individual groups (data in columns), namely *,ɤ, ß, and between individual groups for data in each row (namely letters such as a,b,c ). We are happy to consider an alternative approach but strongly feel that this is the easiest way to document both features of the analysis within a single table.

In table 3, letter b is in all the groups in PPAR, and I think restricted males should be a. The restricted female group has the highest value for UCP1, are you checking individual values looking for outliers?

Authors Response: thank you for spotting this error in superscript lettering which has been corrected. We did check for normality as stated and the associated plots also help identify potential outliers for further testing. Growth restricted females had markedly higher UCP1 expression and there were no statistical outliers within the group or for the fetuses irrespective of birth weight category or sex.

L687-8: Close brackets
L727: APGAR is not previously defined

Authors Response: both corrected, thank you for spotting these omissions.

While revising your submission, please upload your figure files to the Preflight Analysis and Conversion Engine (PACE) digital diagnostic tool, <https://pacev2.apexcovantage.com/>. PACE helps ensure that figures meet PLOS requirements. To use PACE, you must first register as a user. Registration is free. Then, login and navigate to the UPLOAD tab, where you will find detailed instructions on how to use the tool. If you encounter any issues or have any questions when using PACE, please email us at [figures@plos.org](mailto:figures@plos.org). Please note that Supporting Information files do not need this step.

Authors Response: the 3 original figures have been checked as per journal instructions.
